# Transcatheter Palliation of a Young Infant With Obstructed Supracardiac Total Anomalous Pulmonary Venous Connection

**DOI:** 10.7759/cureus.27035

**Published:** 2022-07-19

**Authors:** Mehboob Sultan, Zunaira Zulfiqar, Maryam Khan, Yashfeen Ahmed

**Affiliations:** 1 Paediatric Cardiology, Army Cardiac Centre, Lahore, PAK; 2 Paediatrics, Combined Military Hospital, Lahore, PAK; 3 Internal Medicine, Combined Military Hospital, Lahore, PAK

**Keywords:** pediatric emergency, intervention pediatric cardiology, balloon angioplasty, pulmonary hypertension, total anomalous pulmonary venous connection

## Abstract

Obstructed total anomalous pulmonary venous connection is a life-threatening pediatric cardiac emergency. Infants usually present in critical condition with marked respiratory distress, severe metabolic acidosis, and central cyanosis. Urgent cardiac surgical intervention, despite its high risk, is necessary in order to save the life of the patient. A two-month-old female infant presented to our tertiary care hospital with dense cyanosis and metabolic acidosis. She required mechanical ventilation, but her oxygen saturation did not improve. Her 2D transthoracic echocardiography revealed obstructed supracardiac total anomalous pulmonary venous connection with adequate interatrial communication and severe pulmonary hypertension. After discussion with the family and pediatric cardiac surgical team, it was decided to offer her transcatheter relief of obstructive ascending channel. She underwent successful balloon angioplasty of stenosed levoatrial cardinal vein (vertical vein) with remarkable improvement in blood flow and vessel caliber. She was extubated and her oxygen saturation rose from the high seventies to low eighties immediately after the procedure. She is scheduled for cardiac surgical repair within the next few days. Transcatheter angioplasty is a workable option in stabilizing very sick young infants with obstructed total anomalous pulmonary venous connection, especially supracardiac ones.

## Introduction

Total anomalous pulmonary venous connection is a rare but potentially life-threatening congenital heart defect. It affects roughly 1-3 percent of all children with congenital cardiac disease, and untreated cases have a very high rate of infant death [1,2]. Massive left to right shunt is caused by abnormal drainage of entire pulmonary venous blood to the right atrium directly or through a tributary of the systemic veins [3,4]. Total anomalous pulmonary venous connection is divided into four types based on the location of pulmonary venous drainage into the systemic circulation: supracardiac, infracardiac, cardiac, and mixed.

Patients with unobstructed total anomalous pulmonary venous connection present with signs and symptoms of increased pulmonary flow and congestive cardiac failure along with mild cyanosis. In contrast, patients with obstructive total anomalous pulmonary venous connection present in extremis with severe cyanosis, respiratory distress, and marked metabolic acidosis. The severity of symptoms in total anomalous pulmonary venous connection depends primarily on the degree of pulmonary venous obstruction. The diagnosis is usually established by two-dimensional transthoracic echocardiography.

Surgical management depends on the presence of obstruction, anatomic type, and associated cardiac lesions. Obstructed total anomalous pulmonary venous connection requires urgent surgical intervention [5], whereas unobstructed total anomalous pulmonary venous connection can be dealt with electively. Infants with obstructive total anomalous pulmonary venous connection are the sickest and carry significantly high mortality after surgery. Some of these infants may benefit from transcatheter relief of obstruction before surgery especially very sick, small premature infants with co-morbidities or with isomeric hearts [6-11]. The idea of preoperative stabilization by temporarily relieving the obstruction was developed to improve outcomes for neonatal obstructed total anomalous pulmonary venous connection [12]. We are reporting a case of obstructed supracardiac type total anomalous pulmonary venous connection who was greatly benefited by transcatheter relief of obstructed levoatrial cardinal vein.

## Case presentation

**Figure 1:**
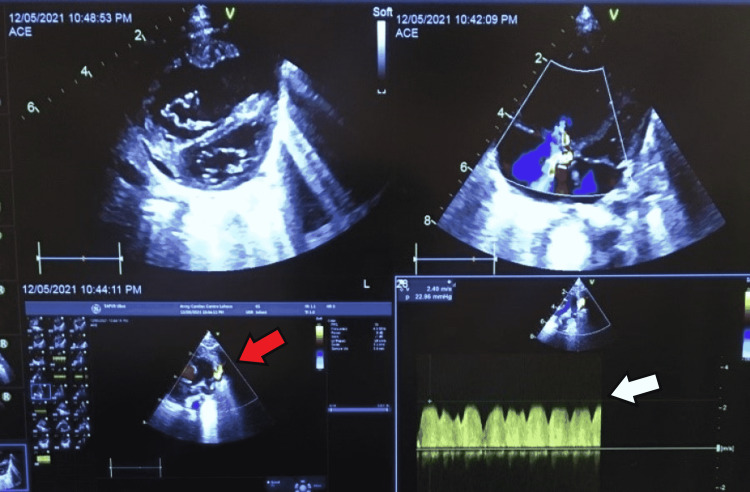
Pre-procedural transthoracic echocardiogram showing dilated right heart, moderate tricuspid regurgitation, and severe pulmonary hypertension. Pulmonary veins draining via ascending levoatrial cardinal vein (vertical vein) into the innominate vein with severe narrowing of the vertical vein (red arrow) and peak pressure gradient of 23 mmHg (white arrow).

A 2.5-month-old girl, born full-term, weighing 3 kg (<5th centile) presented to our hospital with respiratory distress, central cyanosis, and metabolic acidosis. This baby was born at home via spontaneous vaginal delivery in one of the remote areas of Pakistan. No proper medical documentation of length and weight was done at birth but the mother reported the baby looked marginally smaller and continued to fail to thrive over the next couple of months. The baby achieved normal developmental milestones; she remained relatively well for the first two to three weeks of birth but afterward started experiencing gradually increasing tachypnea. The mother also noticed that sometimes her lips turned dusky while crying. They had a few medical checkups locally but the baby was treated on the lines of pneumonia. Referral to the cardiac facility was made around two months of age for murmur and persistent respiratory distress. On examination, she was irritable with acidotic breathing, dusky lips, and cyanosed nails. The liver was palpable 3 cm below the right costal margin and a pan systolic murmur was heard at the mid-left parasternal area. She required mechanical ventilation for acute hypoxemic respiratory failure; however, there was only a marginal improvement in her oxygen saturation. Her arterial blood gas showed a PH of 6.92 with depleted serum bicarbonate and oxygen saturation was 49%. Her transthoracic echocardiography revealed obstructed supracardiac total anomalous pulmonary venous connection with adequate & unrestricted interatrial communication, dilated right atrium & right ventricle, and severe pulmonary hypertension (Figure 1). All pulmonary veins were draining via ascending levoatrial cardinal vein (vertical vein) into the innominate vein and through the superior vena cava to the right atrium. There was severe narrowing at the upper part of the vertical vein with Doppler interrogation showing a continuous flow pattern and peak pressure gradient of 23 mmHg. There was moderate tricuspid regurgitation with a peak pressure gradient of 113 mmHg. After discussion with the family and the pediatric cardiac surgical team, it was decided to offer her emergency transcatheter relief of obstruction in order to stabilize her clinical status.

**Figure 2:**
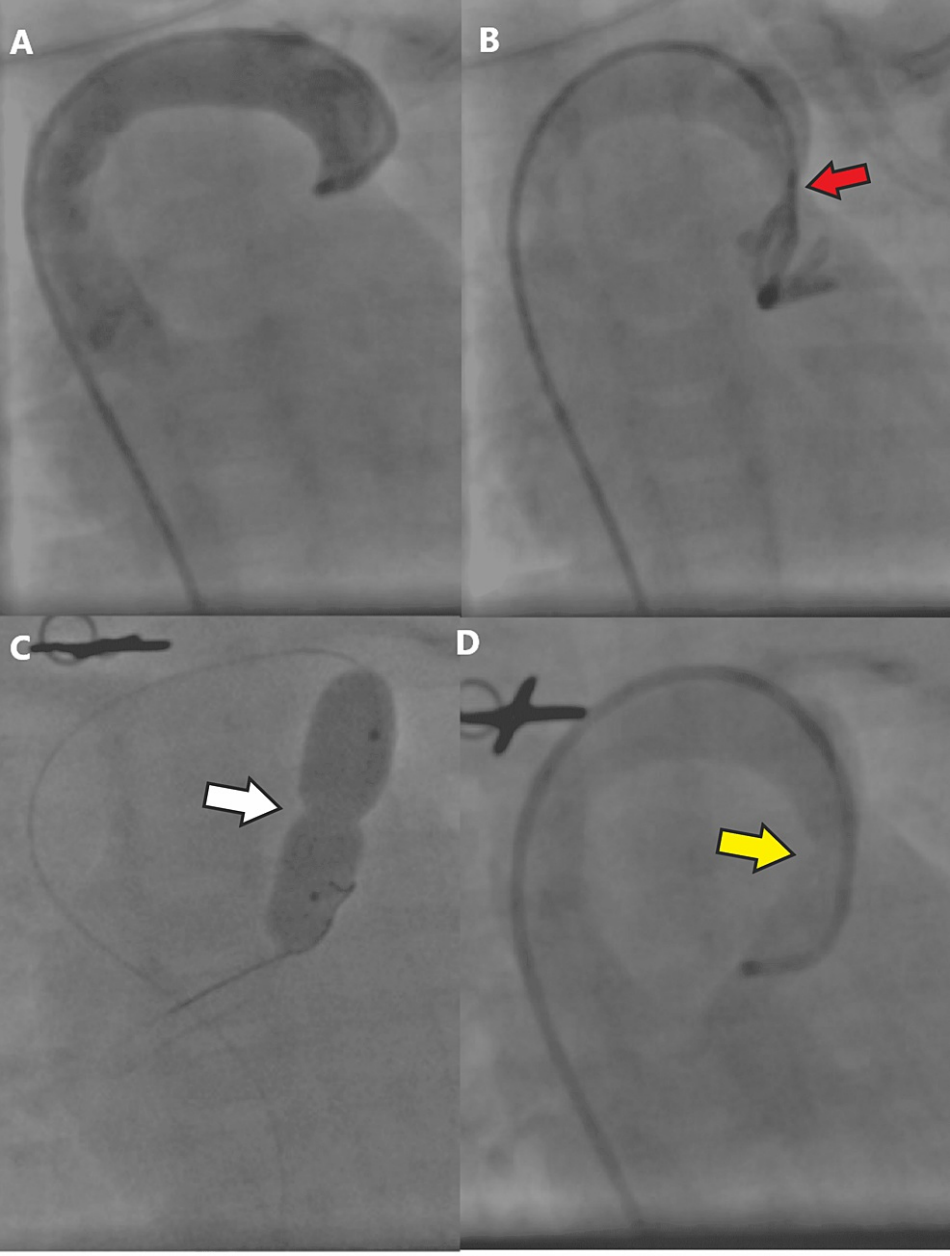
Angioplasty pictures A and B: There was a severe narrowing of the upper part of ascending vertical vein (red arrow). The peak pressure gradient/difference between vertical vein and the innominate vein was 17 mmHg. B and C: Balloon with waist (white arrow) and post dilatation angiogram confirmed improved flow and anatomical caliber of the vessel (yellow arrow).

She was shifted to the cardiac catheterization laboratory on a transport ventilator. Right femoral vein was accessed with a 5F short radial sheath and 5F Judkin right catheter advanced from the femoral vein to the inferior vena cava to the right atrium and into the superior vena cava. The catheter further advanced to the innominate vein and into the vertical vein and hand injection of contrast showed severe narrowing of the upper part of ascending vertical vein. The peak pressure gradient/difference between the vertical and innominate vein was 17 mmHg (Figure 2). All pulmonary veins drained into the common confluence chamber behind the left atrium and drained via ascending vertical vein into the innominate vein to the systemic venous circulation. The stenosed segment of the levoatrial cardinal vein crossed with hydrophilic Terumo wire and exchanged to 0.014 coronary wire, which advanced deep into the right pulmonary artery. We used an 8x20 mm VACS II balloon for gradual dilatation of the stenosed segment. The post-dilatation angiogram showed improved flow and anatomical caliber of the vessel. The option of stenting the vertical vein was not considered as there was a marked improvement in vessel caliber after balloon angioplasty and secondly, early corrective surgery was planned (Figure 2).

**Figure 3:**
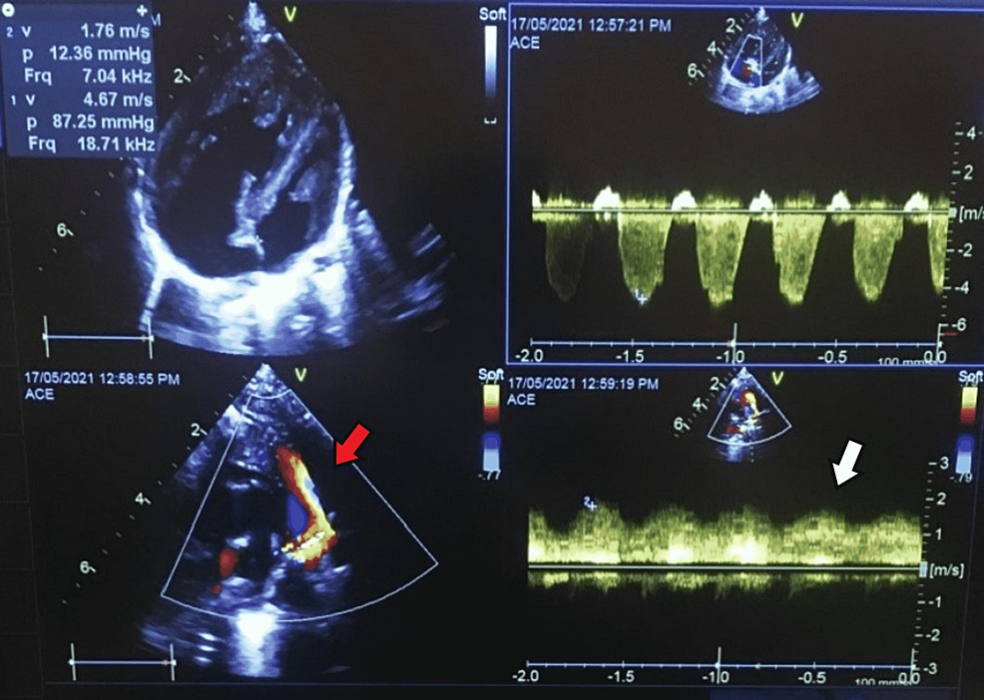
Post procedural transthoracic echocardiogram Improved flow and caliber of ascending levoatrial cardinal vein (red arrow) and peak pressure gradient was around 13mmHg (white arrow) and tricuspid regurgitation gradient was 87mmHg.

Post angioplasty, she was extubated and her oxygen saturation rose to the high seventies immediately after the procedure. Her post-procedural echocardiogram also showed massive improvement (Figure 3). She was gradually weaned off from supplemental oxygen over the next three days and discharged home with a plan to proceed with semi-elective cardiac surgical repair within the next few days (pediatric cardiac surgery will be done at another tertiary care center in Rawalpindi). 

## Discussion

Our patient presented as a life-threatening cardiac emergency with severe metabolic acidosis, profound central cyanosis, and marked respiratory distress, with little response to treatment including mechanical ventilation. Her echocardiography confirmed the diagnosis of obstructed supracardiac total anomalous pulmonary venous connection with severe pulmonary hypertension (Figure 1). Palliative balloon angioplasty improved her clinical status and she was immediately extubated after cardiac catheterization and gradually weaned off from supplemental oxygen inhalation over the next 72 hours. She was planned for surgical repair within the next two weeks as a semi-elective procedure.

Obstructed total anomalous pulmonary venous connection is considered a pediatric cardiac surgical emergency and carries a significantly high operative mortality rate. In surgical management of total anomalous pulmonary venous connection, one of the most important factors adversely affecting surgical outcomes is the presence of an obstruction to pulmonary venous flow. A recent study from Japan analyzed the surgical result of 256 cases of total anomalous pulmonary venous connection operated at a single institute from 1981 to 2016 and reported overall surgical mortality of almost 13% and younger age was reported as a significant risk factor associated with overall postoperative mortality [13]. The overall surgical mortality was exactly 50% for cases with preoperative pulmonary venous obstruction [13]. Another Japanese study shared their surgical experience (1956-2016) of 290 children with total anomalous pulmonary venous connection with a mortality of 24% [14]. Hung et al. published a retrospective experience of 58 cases operated for total anomalous pulmonary venous connection and identified various risk factors responsible for postoperative complications including low body weight, preoperative pulmonary hypertension & pulmonary venous obstruction, emergency surgery, and prolonged cross-clamp time [3].

Preoperative relief of pulmonary venous obstruction is an attractive therapeutic option, especially in supra cardiac total anomalous pulmonary venous connection cases where an obstruction is easily assessable for angioplasty as demonstrated in our case (Figure 2). The idea is not new but due to the rarity of conditions, there is a paucity of reports in the literature. The earliest report of relief of obstructed supracardiac total anomalous pulmonary venous connection was of a three-month-old infant, who underwent successful balloon dilatation of obstructed ascending vein with remarkable hemodynamic improvement, done back in 1985 [6]. In the coming years, various case reports were published using balloons and/or stents in obstructed pathways and included cases of obstructed infra cardiac total anomalous pulmonary venous connection. Published work highlighted the important role of pre-operative angioplasty procedures for obstructive total anomalous pulmonary venous connection in reducing eventual surgical risk [4,7-10,15].

To the best of our knowledge, our case is the first of its kind reported from Pakistan. We used a valvuloplasty balloon with a diameter of 8mm, to dilate the severely narrowed levoatrial cardinal vein with remarkable improvement in vessel caliber and blood flow followed by remarkable hemodynamic recovery. Our case demonstrates how precise assessment and prompt intervention can improve survival for sick children with serious congenital defects. It’s important to remember that the levoatrial cardinal vein is obstructed externally between the left pulmonary artery and left bronchus, thus having the potential of restenosis, and therefore the theoretically better option is to place a stent at the narrow segment. In our case, stent placement was not done as the early surgical intervention was planned.

## Conclusions

Palliative balloon angioplasty for obstructed supracardiac total anomalous pulmonary venous connection is feasible and efficacious in relieving the pulmonary venous obstruction. In high-risk cases with obstructed total anomalous pulmonary venous connection, transcatheter intervention is a lifesaving preoperative option.
